# Clinicopathological characteristics of new primary melanomas in patients receiving immune checkpoint inhibitor therapy for metastatic melanoma

**DOI:** 10.1111/ajd.13807

**Published:** 2022-02-21

**Authors:** Thomas E Pennington, Cathy Yunjia Zhao, Andrew J Colebatch, Pablo Fernandez‐Peñas, Pascale Guitera, Hazel Burke, Richard A Scolyer, Alexander M Menzies, Matteo S Carlino, Serigne Lo, Georgina V Long, Robyn PM Saw

**Affiliations:** ^1^ Melanoma Institute Australia The University of Sydney Sydney NSW Australia; ^2^ Faculty of Medicine and Health The University of Sydney Sydney NSW Australia; ^3^ Royal Prince Alfred Hospital Camperdown NSW Australia; ^4^ Westmead Hospital Westmead New South Wales Australia; ^5^ Department of Tissue Pathology and Diagnostic Oncology Royal Prince Alfred Hospital Sydney NSW Australia; ^6^ New South Wales Health Pathology Sydney NSW Australia; ^7^ Royal North Shore Hospital Sydney NSW Australia

**Keywords:** checkpoint inhibitors, immunotherapy, melanoma, new primary melanoma

## Abstract

**Background:**

Immune checkpoint inhibitors have improved survival in advanced stage melanoma patients. Rates of new primary melanomas (NPM) in patients with prior melanoma have been reported to be as high as 12%. Little is currently known regarding the frequency or characteristics of NPMs occurring in melanoma patients treated with immune checkpoint inhibitors.

**Aim:**

To determine the frequency and describe clinicopathologic characteristics of NPMs diagnosed in patients during or after treatment with immune checkpoint inhibitors for metastatic melanoma.

**Methods:**

A retrospective analysis of prospectively collected data from the Melanoma Institute Australia and Westmead Hospital Dermatology databases. Clinicopathological data for the initial primary melanoma (IPM) and NPM were compared.

**Results:**

Between 2013–2017, 14 NPMs in 13 patients (out of a total of 1047) treated with checkpoint inhibitors were identified. NPMs were significantly thinner than the IPM (median Breslow thickness 0.35 mm *vs* 2.0 mm, *P* = 0.0003), less likely to be ulcerated (0/14 *vs* 6/13, *P* = 0.004) and less likely to have nodal metastases (0/14 *vs* 6/13, *P* = 0.004). NPMs were significantly more likely to be detected in the *in‐situ* stage (6/14 *vs* 0/13, *P* = 0.0016).

**Conclusion:**

NPMs are infrequent in patients treated with checkpoint inhibitors. When they occur, they are usually detected at an early stage and have features associated with a favourable prognosis, most likely reflecting close surveillance. Further study is required to determine long‐term risk in patients achieving a durable response to immune checkpoint inhibitors, and to determine whether the immunotherapy itself influences both their development and biology.

## Introduction

Population‐based studies have demonstrated a significantly increased risk of developing new primary melanomas (NPM) in patients diagnosed with an invasive or *in‐situ* initial primary melanoma.^1^, ^2^, ^3^ One study estimated an odds ratio (OR) of 8.61 for NPM. However, this risk was not stratified according to melanoma stage, and would be difficult to quantify amongst patients with advanced disease in the pre‐immunotherapy era, given the low long‐term survival rates of patients with advanced melanoma. A study from our own database showed cumulative incidence rates of NPMs in patients with stage IV melanoma at 3‐, 6‐ and 12 months of 0.2%, 0.3% and 0.4%, respectively.^4^

Immunotherapy using checkpoint inhibitors has revolutionised therapy for locally advanced and metastatic melanoma patients, with significant improvements in overall survival in phase 3 clinical trials; the combination of anti‐PD1 with anti‐CTLA4 yielding the best results.^5^, ^6^, ^7^ As patients with advanced melanoma are living longer, the risk of developing new primary melanomas will likely increase in this population. It remains unclear whether the incidence and clinicopathological characteristics of NPMs in this population may be altered by the administration of effective drug therapies themselves, particularly immunotherapy. This study sought to document the clinicopathological characteristics of NPMs amongst a cohort of patients with locally advanced or metastatic melanoma treated with immune checkpoint inhibitors.

## Methods

A search of the Melanoma Institute Australia (MIA) and Department of Dermatology, Westmead Hospital databases was performed. Patients with a diagnosis of new primary melanoma during or after treatment of a previous high‐risk resectable stage III (adjuvant treatment) or unresectable stage III/IV (advanced) melanoma with either anti‐CTLA4 (ipilimumab) or anti‐PD1 (nivolumab, pembrolizumab) immunotherapy, or a combination thereof, were included in the analysis. Clinicopathological characteristics of the initial primary melanoma were compared with those of the NPM. In patients with multiple prior primary melanomas, the first (culprit) melanoma (IPM) was determined using the criteria described by Murali and colleagues^8^ The median time from start of immunotherapy to diagnosis of NPM was documented. Statistical analyses were conducted using GraphPad Scientific software. *P* < 0.05 were considered statistically significant. The tumour infiltrating lymphocyte (TIL) grade was derived using the method of Azimi and colleagues.^9^

## Results

Between 2013–17, of 1047 patients receiving immune checkpoint inhibitors, 14 new primary melanomas were detected in 13 patients (cumulative incidence rate 1.3%). The median time from commencement of immunotherapy to diagnosis of the NPM was 322 days. Ten NPMs occurred in males and four in females (2.5:1). The median age at diagnosis of the initial primary melanoma was 67, and 70 for the NPM. Nodal status for the IPMs were clinically node positive in one patient (8%), sentinel node positive in five patients (38%) and sentinel node negative in seven patients (54%). Melanoma stage immediately prior to commencement of immunotherapy was resected stage III in one patient, unresectable stage III in one patient and stage IV in 12 patients. Clinicopathological characteristics of the IPM and NPM are summarised in Table 1.

**Table 1:** Clinicopathological characteristics of IPMs and NPMs

	IPM	Relative incidence (%)	NPM	Relative incidence (%)
Median Breslow thickness	2.0 mm		0.35 mm	
Median clark level	IV		I/II	
Subtype				
SS	6	46	4	29
Nodular	4	31	1	7
LMM	–	–	1	7
MBN	1	8	–	–
Desmoplastic	2	15	–	–
MIS/LM	–	–	6	43
Mucosal	–	–	2	14
Location				
Trunk	7	54	7	50
Head and neck	3	23	2	14
Upper limb	1	8	4	29
Lower limb	2	15	1	7
Ulceration				
Yes	5	38	–	0
No	8	62	14	100
BRAF status				
WT	5	38	2	14
V600E/K	2	15	2	14
Unknown	6	46	10	72
TIL grade				
0	–	–	5	36
1	–	–	0	0
2	–	–	1	7
3	–	–	3	21
Nodal status				
Clinically positive	1	8	–	0
Sentinel node positive	5	38	–	0
Node negative	7	54	14	100
ITMs	2	15	2	14
Stage prior to commencing immunotherapy				
IIIA	1	8	–	–
IIIB	–		–	–
IIIC	1	8	–	–
IIID	–		–	–
IV	11	84	–	–

ITMs, in‐transit metastases; LM, lentigo maligna; LMM, lentigo maligna melanoma; MBN, malignant blue naevus; MIS, melanoma *in situ*; SS, superficial spreading; WT, wild type.

The mean Breslow thickness of the initial primary melanoma was 2.9 mm (range 0.8–5.4 mm) *versus* 0.8 mm (range 0–3 mm) for the new primary melanoma (*P* = 0.0003). Ulceration was present in 6/13 IPMs (46%) compared to 0/14 NPMs (0%), (*P* = 0.004). There were more NPMs diagnosed on the upper limb (4/13 *vs* 1/14, *P* = 0.11) but there was no statistically significant difference in tumour location between the two groups. NPMs had a statistically significantly lower T stage according to the American Joint Committee on Cancer (AJCC) 8^th^ Edition staging criteria^10^ compared with the IPM (*P* = 0.0003). BRAF mutation status was known in 7/13 IPMs and 5/14 NPMs. The BRAF V600E mutation was present in 2/7 IPMs and 3/5 NPMs (*P* = 0.3198). BRAF status was not ascertained in the NPMs of those patients who had a BRAF mutation in their IPM. However, in two of the three NPMs with a BRAF V600E mutation, their IPM was BRAF wild‐type, and in the third, the BRAF status was unknown.

The median follow‐up from diagnosis of the IPM was 34.8 months (range 8–204 months), and 6 months from diagnosis of the NPM (range 1.8–34 months).

### Nodal status

6/13 patients had either clinically detected nodal metastases or micrometastasis to the sentinel node related to their initial primary melanoma. None of the 14 new primary melanomas underwent sentinel node biopsy (SNB) or node dissection surgery, although 3/14 NPMs were otherwise eligible for SNB based on contemporary Breslow Thickness criteria. Therefore, node status specific to these NPMs is unknown, albeit all 14 were clinically node negative. There was no evidence of new nodal metastasis to a nodal basin draining the NPM on subsequent imaging investigations. Two patients developed in‐transit metastases from their IPM and two separate patients had in‐transit metastases recorded in association with their NPM (see Discussion).

### Distant metastasis

Seven patients (54%) had distant metastasis at the time of diagnosis of the initial primary melanoma or shortly afterwards, and a further four patients (total 85%) had distant metastasis just prior to commencement of immunotherapy. One patient (8%) was stage IIIA and received adjuvant pembrolizumab on trial, and one patient was classified as unresectable stage IIIC.

### Immune checkpoint inhibitor schedule

Nine patients received first‐line anti‐PD‐1 monotherapy, one patient received first‐line anti‐CTLA‐4 monotherapy and four patients received combination therapy with anti‐PD‐1/anti‐CTLA‐4. One patient on anti‐PD‐1 monotherapy also received a BRAF/MEK inhibitor. Immunotherapy was ceased in two patients for serious adverse events. One of these patients was re‐challenged with anti‐PD‐1 monotherapy 9 months later for disease progression. Eleven patients continued on anti‐PD‐1 monotherapy after the new primary melanoma diagnosis, whilst one patient continued on combination immunotherapy. Two patients also received palliative radiotherapy for symptomatic metastases.

### Response to treatment

There were six *in‐situ*, and eight invasive, new primary melanomas detected whilst on immunotherapy amongst the 13 patients. There were no clinically detected nodal macrometastases at the time of diagnosis of the NPM (0/14). There were no sentinel node biopsies performed as part of management of the three otherwise‐eligible NPMs (0/3). Two patients had in‐transit metastases (ITM) in the lymphatic drainage distribution of the NPM, compared with two ITMs related to initial primary melanomas (*P* = 0.638). On RECIST criteria,^11^ four patients (31%) were assessed to have had a complete response to immunotherapy, five patients a partial response (38%), two patients had stable disease (13%) and two patients had disease progression (13%). Of the three patients with NPMs of T‐stage 2 or higher, one had had a complete response to immunotherapy, one had had a partial response and one had stable disease. Twelve patients were alive at last follow‐up, and one patient (8%) had died of advanced melanoma.

## Evidence of immune effect on new primary melanomas

Of the nine invasive new primary melanomas, five (36%) displayed no tumour‐infiltrating lymphocytes (TILs), one (7%) displayed a TIL grade 2 infiltrate, and three (21%) had TIL grade 3 infiltrates. TIL assessment was not available for the initial primary melanomas.

## Discussion

This study presents data documenting our experience with new primary melanomas in patients treated with immune checkpoint inhibitors for a prior high risk or metastatic melanoma. Whilst patient numbers are small, it appears clear that NPMs in this cohort are uncommon and tend to be diagnosed at an earlier stage. The median Breslow thickness of NPMs in our cohort was 0.35 mm. This compares favourably with previous studies in patients with stage I and II melanoma showing most NPMs are detected with a Breslow thickness <1 mm.^12^ It is likely that this can be explained by close follow‐up, or “lead‐time bias”. However, another plausible explanation might be immune modulation secondary to a checkpoint‐inhibitor effect on an evolving NPM. Our cohort is too small to confidently support or refute this hypothesis, but it remains an intriguing potential effect of immunotherapy. The estimated cumulative incidence of NPMs of 1.3% at 5 years cannot be easily compared to previous cohorts. However, it is noted that a study from our institution estimated a 1‐year cumulative incidence of NPMs in advanced stage III and IV melanoma patients of 0.4%, in the pre‐checkpoint inhibitor era.^4^ We will probably not be able to directly compare long‐term incidence rates amongst advanced melanoma patients in the pre‐ and post‐checkpoint inhibitor era, particularly as patients with advanced melanoma did not undergo regular skin surveillance due to their poor prognosis in an era before effective systemic treatments. But it will be intriguing to note whether incidence rates are significantly different compared to the rate of NPMs in earlier stage disease. This should be a subject of future research.

It is difficult to accurately assess whether immune checkpoint inhibitors reduce the *incidence* of new primary melanomas, as any estimates carry inherent biases. For example, patients may at times have their NPM detected by their Primary Care Physician, Dermatologist or other clinician, and these lesions may not get recorded in databases. Furthermore, as all 13 patients had an original primary cutaneous melanoma, it is possible these NPMs represent missed synchronous melanomas, that have only been picked up subsequently at follow‐up.

Additionally, it is difficult to conclude whether there is a difference in the rate of new primary melanomas amongst patients with differing responses to immunotherapy. Stage IV patients who do not respond to immunotherapy are unlikely to live long enough to develop a new primary melanoma. The vast majority of long‐term survivors of stage IV melanoma are immunotherapy responders, creating an inherent selection bias. It can be inferred that responders to immunotherapy are more likely to live long enough to develop an NPM than non‐responders. However, even amongst this cohort, NPMs are not common.

It is interesting to examine the rate of concordance of BRAF mutation amongst initial and new primary melanomas, in patients on immunotherapy. Whilst BRAF mutations were not routinely ascertained for all patients in this cohort, two of the three NPMs with BRAF mutation had BRAF *wild‐type* IPMs. A more comprehensive assessment will be required to determine whether there is a higher rate of BRAF mutation in NPMs of patients who have had checkpoint inhibitor immunotherapy.

Surrogate markers of immunologic modulation of new primary melanomas may be histologic features such as regression and tumour‐infiltrating lymphocytes. Melanoma metastases that respond to immunotherapy have demonstrated increased CD8+ and CD3+ lymphocyte counts at the invasive tumour margin.^13^, ^14^, ^15^, ^16^, ^17^ If this effect could be demonstrated in NPMs, the hypothesis that the immunotherapy can reduce the incidence of NPMs would gain traction. The majority of NPMs in our cohort had no TILS (67%), but the work of TILs might be to eradicate evolving melanomas before they are detected clinically, and hence the true effect of immunotherapy on the incidence of NPMs might only be demonstrated by a reduction in the incidence of NPMs. This will require much longer follow‐up and larger cohorts to reliably establish. As more “early stage” melanoma patients are receiving adjuvant immunotherapy, it may be easier to observe a change in incidence of NPMs in this group, who inherently live longer and develop more NPMs.

Subgroup analysis shows 2/6 patients who had no TILs in their new primary melanoma had minimal response (stable disease) to immunotherapy, whereas all four (4/4) patients with grade 2 or 3 TILS in the NPM had partial or complete response to immunotherapy for the initial metastatic melanoma. This is a highly selected group, as stage IV patients with no response to immunotherapy are unlikely to survive long enough to develop an NPM. It is also difficult to entirely exclude distant metastasis from the NPM in the presence of previously established metastases. It is possible that disease progression is occasionally due to metastasis from a biologically resistant NPM.

The distribution of melanomas by body site was similar amongst initial and new primary melanomas, although there was a larger number of NPMs detected on the upper limbs (4 *vs* 1). Whether immunotherapy alters the distribution of NPMs remains to be seen with larger data sets. One theory would be that NPMs arising in areas of high UV exposure may be halted more effectively than those with a low UV signature, which may have an effect on the distribution of NPMs compared to IPMs in this cohort.

Similarly, there was no difference between groups in terms of the presence of in‐transit metastases (two patients developed ITMs related to their initial primary melanoma, and two different patients developed ITMs in relation to their new primary melanoma). Given that both patients who were recorded to have ITMs related to their NPM had T1a lesions (Breslow thickness 0.7 mm and 0.4 mm respectively), it seems more likely these ITMs were in fact distant dermo‐subcutaneous metastases related to the IPM, than true NPM ITMs.

Despite the uncertainties raised by our early data, it does appear new primary melanomas are detected at an earlier stage than their predecessor lesion and therefore are inherently lower risk than the first melanoma. Longer term data in larger population groups is lacking, and the effect of immunotherapy on the incidence and characteristics of NPMs is a subject requiring future research. It remains evident that the cumulative incidence of NPMs in long‐term survivors of high risk and metastatic melanoma who have undergone checkpoint inhibitor immunotherapy is low, but these patients still require close and ongoing skin surveillance.

## Conflict of Interest

SL, RPMS supported by Melanoma Institute Australia. RPMS has received honoraria for advisory board participation from MSD, Novartis and Qbiotics and speaking honoraria from BMS. RAS has received fees for professional services from Qbiotics, Novartis, Merck Sharp & Dohme, NeraCare, AMGEN Inc., Bristol‐Myers Squibb, Myriad Genetics, GlaxoSmithKline. GVL is consultant advisor for Aduro Biotech Inc, Amgen Inc, Array Biopharma inc, Boehringer Ingelheim International GmbH, Bristol‐Myers Squibb, Highlight Therapeutics S.L., Merck Sharpe & Dohme, Novartis Pharma AG, QBiotics Group Limited, Regeneron Pharmaceuticals Inc, SkylineDX B.V. PFP has reported advisory role for Janssen, Roche, AbbVie, Merck Sharp & Dohme, Sun Pharmaceuticals, Merck, Novartis, Sanofi, Leo and Lilly, and he has conducted clinical trials for Arena, Akaal Pharma, Xoma, Kyowa Hakko Kirin, AbbVie, OncoSec, Lilly, CSL Behring, Boehringer Ingelheim, Pfizer, mRage, Eisai, Jiansu Hengrui, Bristol‐Myers Squibb, Sun Pharma, Novartis, Roche, Galderma, Regeneron, UCB, GlaxoSmithKline and Amgen.

## References

[1] McCaul KA , Fritschi L , Baade P *et al*. The incidence of second primary invasive melanoma in Queensland, 1982–2003. Cancer Causes Control 2008; 19: 451–8. 18167620 10.1007/s10552-007-9106-5

[2] Youlden DR , Youl PH , Soyer P *et al*. Distribution of subsequent primary invasive melanomas following a first primary invasive or in situ melanoma Queensland, Australia, 1982–2010. JAMA Dermatol. 2014; 150: 526–34. 25093216 10.1001/jamadermatol.2013.9852

[3] Giles G , Staples M , McCredie M *et al*. Multiple primary melanomas: an analysis of cancer registry data from Victoria and New South Wales. Melanoma Res. 1995; 5: 433–8. 8589618

[4] Zimmer L , Haydu LE , Menzies AM *et al*. Incidence of new primary melanomas after diagnosis of stage III and IV melanoma. J. Clin. Oncol. 2014; 32: 816–23. 24297954 10.1200/JCO.2013.49.5572

[5] Wolchok JD , Chiarion‐Sileni V , Gonzalez R *et al*. Overall survival with combined nivolumab and ipilimumab in advanced melanoma. N. Engl. J. Med. 2017; 377: 1345–56. doi:10.1056/NEJMoa1709684 28889792 PMC5706778

[6] Ribas RC , Schachter J , Arance A *et al*. Pembrolizumab versus ipilimumab in advanced melanoma (KEYNOTE‐006): post‐hoc 5‐year results from an open‐label, multicentre, randomised, controlled, phase 3 study. Lancet Oncol. 2019; 20: 1239–51. doi:10.1016/S1470-2045(19)30388-2. Epub 2019 Jul 22. 31345627

[7] Schachter RC , Long GV , Arance A *et al*. Pembrolizumab versus ipilimumab in advanced melanoma. KEYNOTE‐006 investigators. N. Engl. J. Med. 2015; 372: 2521–32. doi: 10.1056/NEJMoa1503093. Epub 2015 Apr 19. PMID: 25891173. 25891173

[8] Murali R , Brown PT , Kefford RF *et al*. Number of primary melanomas is an independent predictor of survival in patients with metastatic melanoma. Cancer 2012; 118: 4519–29. 22736239 10.1002/cncr.27693

[9] Azimi F , Scolyer RA , Rumcheva P *et al*. Tumour‐infiltrating lymphocyte grade is an independent predictor of sentinel lymph node status and survival in patients with cutaneous melanoma. J. Clin. Oncol. 2012; 30: 2678–83. 22711850 10.1200/JCO.2011.37.8539

[10] Amin MB , Edge S , Green F *et al*. AJCC Cancer Staging Manual, 8th edn. Chicago, IL: Springer International Publishing: American Joint Commission on Cancer, American College of Surgeons, 2017. ISBN 978‐3‐319‐40617‐6.

[11] Eisenhauer EA , Therasse P , Bogaerts J *et al*. New response evaluation criteria in solid tumours: revised RECIST guideline (version 1.1). Eur. J. Cancer 2009; 45: 228–24716. 19097774 10.1016/j.ejca.2008.10.026

[12] Schoellhammer HF , Torisu‐Itakura H , Huynh Y *et al*. Second primary melanoma: risk factors, histopathologic features, and survival. J. Clin. Oncol. 2009; 27 (15 suppl): 9073–3. PMC5555365 27779995

[13] Vilain RE , Menzies AM , Wilmott JS *et al*. Dynamic changes in PD‐L1 expression and immune infiltrates early during treatment predict response to PD‐1 blockade in melanoma. Clin. Cancer Res. 2017; 23: 5024–3317. 28512174 10.1158/1078-0432.CCR-16-0698

[14] Gide TN , Quek C , Menzies AM *et al*. Distinct immune cell populations define response to anti‐PD‐1 monotherapy and anti‐PD‐1/anti‐CTLA‐4 combined therapy. Cancer Cell 2019; 35: 238–255.e6. doi:10.1016/j.ccell.2019.01.003 30753825

[15] Tumeh PC , Harview CL , Yearley JH *et al*. PD‐1 blockade induces responses by inhibiting adaptive immune resistance. Nature 2014; 515: 568–71. 25428505 10.1038/nature13954 PMC4246418

[16] Taube JM , Klein A , Brahmer JR *et al*. Association of PD‐1, PD‐1 ligands and other features of the tumor immune microenvironment with response to anti‐PD‐1 therapy. Clin. Cancer Res. 2014; 20: 5064–74. doi:10.1158/1078-0432.CCR-13-3271. Epub 2014 Apr 8. 24714771 PMC4185001

[17] Shields BD , Mahmoud F , Taylor EM *et al*. Indicators of responsiveness to immune checkpoint inhibitors. Sci. Rep. 2017; 7: 807 doi:10.1038/s41598-017-01000-2 28400597 PMC5429745

